# A Real-Time Wearable Physiological Monitoring System for Home-Based Healthcare Applications

**DOI:** 10.3390/s22010104

**Published:** 2021-12-24

**Authors:** Jin-Woo Jeong, Woochan Lee, Young-Joon Kim

**Affiliations:** 1 Department of Electronic Engineering, Gachon University, Seongnam 13120, Korea; jason180596@gachon.ac.kr; 2 Department of Electrical Engineering, Incheon National University, Incheon 22012, Korea

**Keywords:** ECG/EMG sensing, physiological monitor, smart wearable device, wireless communication, rehabilitation training

## Abstract

The acquisition of physiological data are essential to efficiently predict and treat cardiac patients before a heart attack occurs and effectively expedite motor recovery after a stroke. This goal can be achieved by using wearable wireless sensor network platforms for real-time healthcare monitoring. In this paper, we present a wireless physiological signal acquisition device and a smartphone-based software platform for real-time data processing and monitor and cloud server access for everyday ECG/EMG signal monitoring. The device is implemented in a compact size (diameter: 30 mm, thickness: 4.5 mm) where the biopotential is measured and wirelessly transmitted to a smartphone or a laptop for real-time monitoring, data recording and analysis. Adaptive digital filtering is applied to eliminate any interference noise that can occur during a regular at-home environment, while minimizing the data process time. The accuracy of ECG and EMG signal coverage is assessed using Bland–Altman analysis by comparing with a reference physiological signal acquisition instrument (RHS2116 Stim/Recording System, Intan). Signal coverage of R-R peak intervals showed almost identical outcome between this proposed work and the RHS2116, showing a mean difference in heart rate of 0.15 ± 4.65 bpm and a Wilcoxon’s *p* value of 0.133. A 24 h continuous recording session of ECG and EMG is conducted to demonstrate the robustness and stability of the device based on extended time wearability on a daily routine.

## 1. Introduction

The prevention of ischemic stroke and stroke recurrence is an important public health concern. Nearly 25% of strokes in the United States are recurrent strokes and stroke causes approximately 1 in 20 deaths [1]. As electrocardiography is one of the most important physiological signals for cardiovascular health and the autonomic nervous system (ANS), cardiac monitoring has been proven to demonstrate relevance to stroke. Several ECG studies have been reported the quantitative ECG measurements in clinical applications to evaluate the relationship between cardiac, neurological, and functional outcomes of ischemic stroke [2,3]. After the strike of stroke, survivors often suffer from hemiplegia which highly affects their daily activities [4]. Hemiplegia generally reveals asymmetrical deficits in gait and is one of the most common disabilities observed in the post-stroke phase. Asymmetrical gait can result from muscle weakness, leading to incompetent mobility, lack of balance, and the threat of muscular wounds to healthy limbs [5,6]. Post-stroke recovery depends on neural adaptation and task-specific repetitive exercise according to the basics of neuroplasticity [7]. Neurorehabilitation training has been widely adopted to reduce the disability caused by the stroke [8] and various forms of neurorehabilitation including EMG-based robotic or visual support have been investigated [9,10,11].

In order to perform ECG analysis and EMG signal-based motor recovery training, traditional Holter monitors and wired EMG monitor devices are applied to patients upon clinical visits. Unfortunately, intermittent ECG abnormalities can go undetected by an on-visit examination [12] and rehabilitation therapy in dedicated facilities for a prolonged period increases the cost and the limited number of therapists results in the delay of treatment, restricting the patient from taking the best advantage of the critical time-limited opportunity [13,14]. Therefore, a wearable home-based monitoring device that is capable of providing immediate medical feedback and relevant ambulatory action is essential. Further, when EMG signals are used for rehabilitation training, the delay in data processing should be minimized while wirelessly transmitting [9,10,11]. Hence, a real-time user-friendly physiological monitoring device and platform that enables remote medical care and patient-driven recovery training is necessary.

Various forms of physiological monitoring devices are commercially available but most of them are designed for recreational purposes. Most of recently developed monitoring devices lack the compactness [15,16,17,18,19,20,21] and wireless connectivity [22] which is essential for everyday wearable application. Other monitoring devices are limited to a single ECG or EMG channel [15,17,23,24] and a long-term stability test including the interference study is left out [15,17,18,24]. Furthermore, smart sensor networks that utilize a cloud network environment and machine learning have been proposed by various research groups [25,26,27] but existing personal healthcare monitoring devices fail to demonstrate the wearable monitoring platform with user-friendly personal smartphone connectivity features for real-time monitoring [18] and cloud networking for further data processing [15,16,17,22,23,24]. 

In this study, a wearable continuous ECG and EMG monitoring system for real-time detection is proposed. The developed system consists of three major sub-systems: (1) a wearable wireless physiological (ECG and EMG) signal monitoring device, (2) a host device (Android smartphone) with real-time monitoring and data processing software, and (3) in-depth bio-signal analysis through a cloud network server. The analog circuit and wireless telemetry for the ECG/EMG data collection are fabricated on a compact printed circuit board (PCB) and encapsulated in a robust housing unit for enhanced wearability. The physiological data are wirelessly transmitted to a smartphone or a laptop for real-time signal monitoring, recording, and data analysis via BLE, allowing users or therapists to access the ECG/EMG information in real-time plot. The frequency spectrum is checked once in every minute to detect the level of interference and it is utilized to adaptively determine the order of the digital filter, optimizing the delay noise. The accuracy of this work is assessed using a reference precision instrument, showing an excellent match. The stability of the developed physiological monitoring system has been demonstrated by a 24 h continuous recording session with daily activity and further biopotential data were processed in a network server for a heartbeat per minute (BPM), heart-rate variability (HRV) and muscle activity monitoring. The key contributions of this paper are as follows.

- The proposed surface bio-potential acquisition system is compactly devised for an everyday wearable application with supporting long-term stability validation. The total area of the monitoring device is just about the size of a button-shape battery (CR2032). The overall system implementation is cost effective compared to existing systems with a dedicated host device.
- A host node manages the role of the monitoring device and displays the signals in a real-time plot. Furthermore, a healthcare network is established between the host node and a cloud server where an intelligent analysis is performed, and remote clinical support can be provided by the physicians.
- A short-term physiological signal acquisition session with a reference instrument and series of analyses concludes that the signal quality of this work is precise. A practical long-term ECG and EMG acquisition session verifies the feasibility and wearability of the proposed device under a regular daily activity, including aggressive exercise.

The rest of this paper is organized as follows. Section 2 presents the design and fabrication of the monitoring device, followed by a discussion of the role and function of the host node. Section 3 provides the experimental results. Finally, Section 4 concludes this paper and suggests future work as a discussion. 

## 2. Design of the Proposed System

For wearable monitoring applications, a small-size and light-weight data acquisition device is desired. To further enhance the patient’s comfort and mobility, the physiological data are wirelessly transmitted to a host smartphone for real-time monitoring. The ubiquity of the internet and smartphones can support remote clinical participation, but home-based physiological signal acquisition devices are vulnerable to external noise in an everyday environment, especially the powerline interference (PLI) [28,29,30]. Here, we present the problem in an everyday environment and provide methods to overcome the issue. 

**Figure 1:**
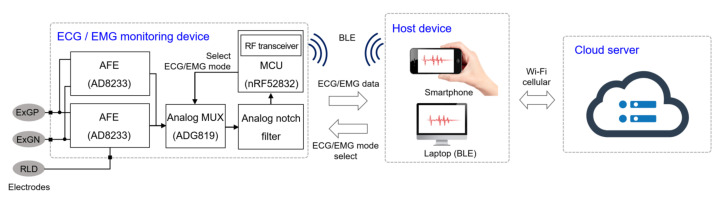
Block diagram of the monitoring system. The biopotentials are acquired from the electrodes and amplified by the analog front-end (AFE). The analog signals are sampled and wirelessly transmitted to the host device, where the signal is reconstructed in real-time for monitoring. This data are saved in the local device and stored in a cloud server via Wi-Fi or cellular communication.

A software platform for an Android device is implemented for data processing and recording where the patient can monitor their bio-signals in real time through their personal smartphone. The software also detects the noise level of the incoming signal, and an adaptive digital filter is implemented to remove unwanted interference, without introducing unnecessary process delay. The recorded data can be distributed to a therapist on a cloud server followed by further data analysis. The block diagram of the overall system is shown in Figure 1.

### 2.1. Monitoring Device Design and Fabrication

**Figure 2:**
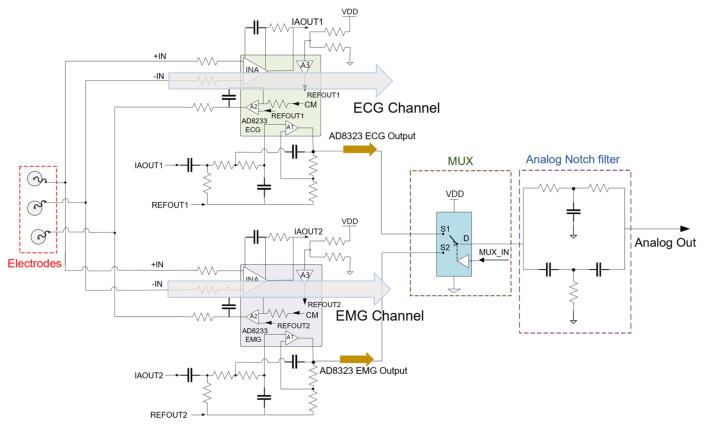
Schematic of the analog front-end. Amplification channel is selected from the analog multiplexer (MUX) from the microcontroller. The 2nd-order analog notch filter eliminates the powerline interference and the analog signal is delivered to the ADC for sampling.

To ensure reliable ECG and EMG capture, the analog front-end (AFE) modifies the analog biopotentials with an instrumentation amplifier configured to amplify the target signals and attenuate common-mode signals. We realized the dual signal capturing system by splitting the electrode node into two AFE channels for both ECG and EMG applications. The input impedance of the AFE is larger than 10 GΩ and the dual-channel configuration introduces a slight gain reduction of less than 10%. A second-order high-pass filter and an amplified second-order low-pass filter conditions the incoming biopotential signal and a right-leg drive circuit drives the common-mode voltage at the electrodes to further enhance the common-mode rejection. We implemented an analog notch filter (twin-T) prior to digital filters, since high order digital filters introduce signal distortion [31] and require a large amount of calculation, which is not suitable for a real-time monitoring system. An analog multiplexer controlled by the microcontroller enables channel selection. The schematic and specifications of the AFE is shown in Figure 2 and Table 1, respectively. 

**Figure 3:**
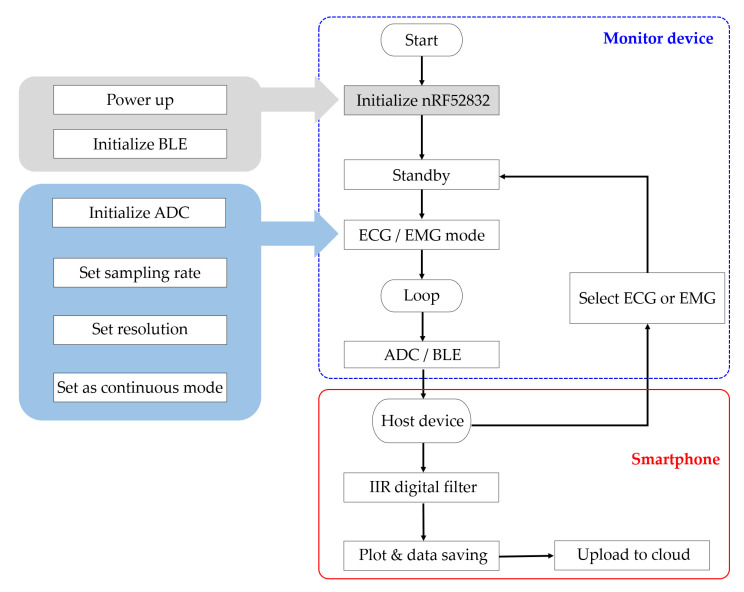
Flow chart of the proposed ECG monitoring system. Upon power up, the microcontroller initializes the BLE for standby. The host device pairs with the monitor device for mode selection (ECG, EMG). Once the measurement mode has been selected, the monitor device samples the incoming analog signal and transmits the data to the host device. The host device performs an additional filtering and displays a real-time plot. The data are saved in the local device and uploaded to cloud server for further analysis.

The microcontroller with integrated analog-to-digital converter (ADC) then samples the biopotential signal and processes for BLE transmission. The signal is sampled at 10 kSps for accurate signal reconstruction and the microcontroller conditions the signal for amplitude and reference level. To reduce the power consumption, the data are stored in a buffer and the BLE transmits in a burst mode every 24 ms. The microcontroller of the device also optimizes power consumption by coordinating system activation, wake, sleep, and power down. Once the system is initialized, active components awaken only when in use and enter a low-power state otherwise. Upon initial power on, the microcontroller is initialized, and the device waits for a mode selection. Once the device is paired with an external host BLE device and a mode selection command is given, the ADC is initialized, and the radio transmits the corresponding data via BLE. From the mode command, the microcontroller drives an output pin to the multiplexer for relevant signal acquisition (Figure 3).

**Figure 4:**
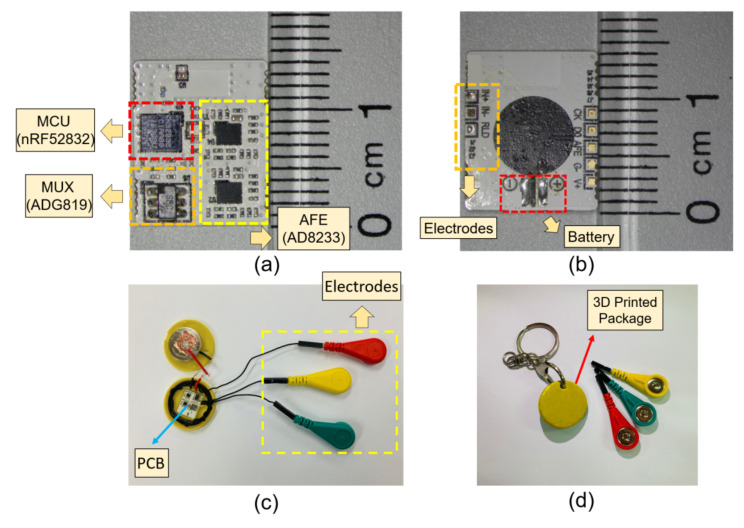
Images of the fabricated device. (**a**) Top view of the populated PCB; (**b**) bottom view of the PCB; (**c**) image of the device encapsulated in a button shaped container with the cap open; (**d**) the cap of the container is closed, ready for use.

The circuit components are populated on a 14 mm × 10 mm × 0.5 mm sized printed circuit board (PCB) using soldering paste (TS391LT, Chip Quik, Ancaster ON, Canada), including the chip-scale ICs and antenna. To make the monitor device suitable for everyday activity, the PCB is encapsulated in a 3D printed button-shape housing with a battery (CR2032, Panasonic, Kadoma, Japan). The wires for electrode interconnection are soldered on to the PCB and a soft layer of PDMS (Sylgard 184, Dow Corning, Midland MI, USA) is molded to the PCB and the wires to prevent electrical shorts and accidental wire breakage. The battery is fixed on the cap of the button-shape housing, which is designed as a twist-lock for an easy battery replacement. The wires are wound around the housing for an adjustable wire length configuration for various monitoring applications (Figure 4). The overall cost of the prototype device fabrication, including the PCB and circuit components, is estimated around USD 40. The proposed device is compatible with a typical Bluetooth 4.0 enabled Android device, where other existing work requires a dedicated host device [17,18].

### 2.2. Host Node Software Implementation

The goal of the software platform is to provide comfort and easy monitoring for the patient and the therapist. Therefore, most of the work is implemented in an application on an Android platform. Once the host device pairs with the monitor device, it receives the physiological data through BLE and feeds it into a real-time infinite impulse response (IIR) digital filter which eliminates any remaining noise signal. Once in every minute, the software detects the magnitude of the noise signal by taking a FFT of the signal in the past 24 ms and this information is used to adaptively determine the order of the IIR filter. In this way, the amount of computation can be reduced, and any unnecessary time delay caused by the calculation can be optimized. The data are reconstructed and visualized into a real-time plot for on-the-spot monitoring. The software platform then logs the ECG/EMG data into the local folder every 24 ms, which is synchronized to a Health Insurance Portability and Accountability Act- (HIPAA) compliant network server every 5 min for further analysis and remote clinical support. 

### 2.3. Data Analysis on Remote Server

Once the file is updated to the server, a machine-learning algorithm can further process the ECG information for R-peak and atrial fibrillation (AF) detection. For sake of demonstration, we present a set of data analysis based on the R-peak detection, followed by a calculation of HRV and BPM. For the EMG data analysis, the raw real-time EMG signal is rectified and integrated to quantify the muscle activity to model the patient’s average activity.

## 3. Experimental Results

### 3.1. Continuous ECG and EMG Monitoring 

**Figure 5:**
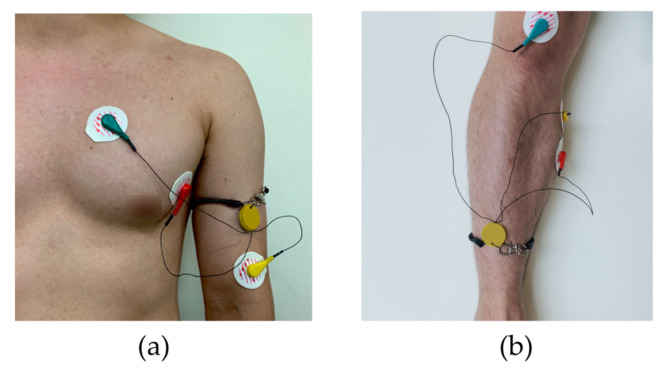
The measurement setup and electrode location. (**a**) The ECG electrodes were placed in a lead II orientation; (**b**) EMG electrodes are placed to measure the surface EMG signals from activation signal at the right medial gastrocnemius muscle.

**Figure 6:**
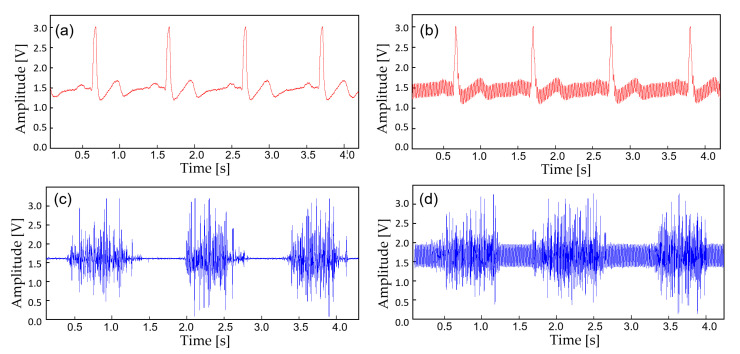
A noise comparison with the captured biopotential signals. (**a**) ECG acquired outdoors; (**b**) ECG at 30 cm away from the wall power; (**c**) EMG acquired outdoors; (**d**) EMG at 30 cm away from the wall power.

**Figure 7:**
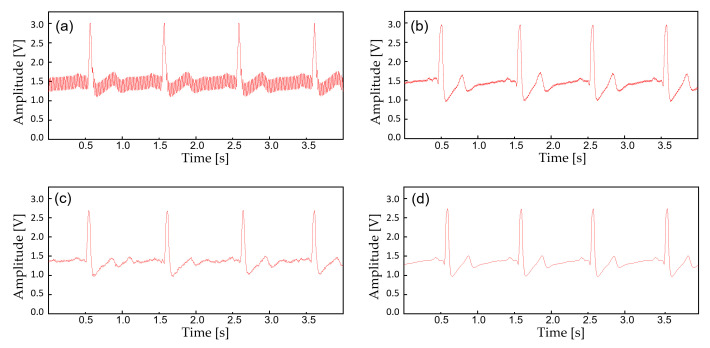
The biopotential signals with additional filtering. (**a**) Raw ECG acquired from the AFE at 30 cm away from the wall power; (**b**) ECG filtered with an analog notch filter (twin-T); (**c**) ECF filtered with an IIR digital filter; (**d**) ECG filtered with (**b**,**c**).

In this study, the monitoring device is worn with a commercial Ag/AgCl (2223H, 3M, St. Paul MN, USA) medical-grade electrodes (Figure 5). The ECG electrodes were placed in a lead II orientation and the EMG electrodes are placed to measure the surface EMG signals from activation signal at the right medial gastrocnemius muscle. The device is tightly fastened to the limb with an adjustable strap and a ring. Signal filtering is unnecessary when the patient is outdoors or in a room with no noise source. But when the subject is indoors with typical consumer electronics and power source nearby, significant interference occurs (Figure 6). The second order low-pass filter (cutoff at 41 Hz) of the AFE for ECG is not able to eliminate the power supply noise (Figure 7a) and the noise is worse for EMG since the biopotential signal bandwidth contains the mains frequency. Therefore, a second order twin-T notch filter centered is implemented to further reduce the power line interference [32] (Figure 7b). 

**Figure 8:**
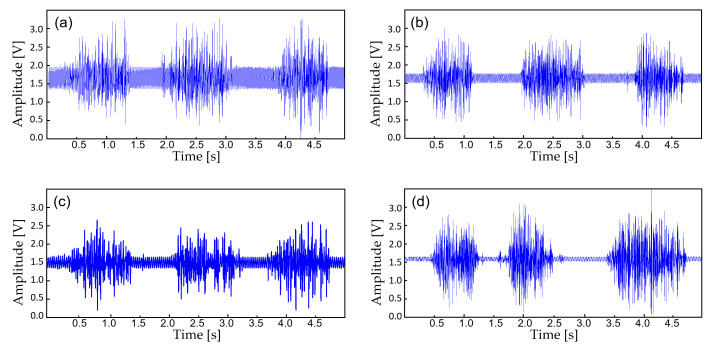
The biopotential signals with additional filtering. (**a**) Raw EMG acquired from the AFE at 30 cm away from the wall power; (**b**) EMG filtered with an analog notch filter (twin-T); (**c**) EMF filtered with an IIR digital filter; (**d**) EMG filtered with (**b**,**c**).

**Figure 9:**
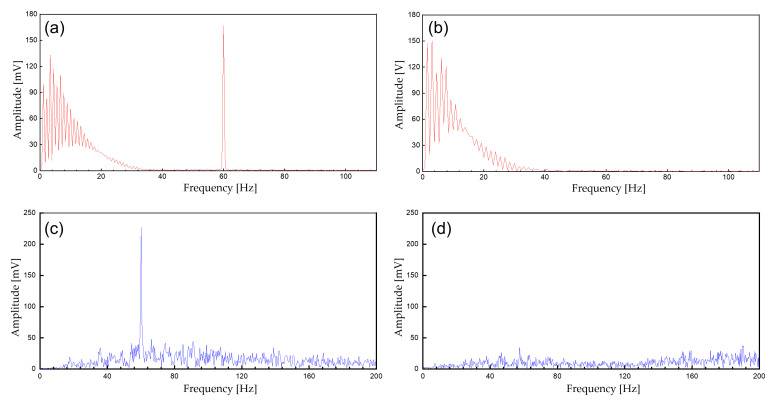
The spectrum of the biopotential signal. (**a**) Unfiltered ECG signals; (**b**) filtered ECG signals (analog notch and digital IIR filter); (**c**) unfiltered EMG; (**d**) filtered EMG signals (analog notch and digital IIR filter).

The device transmits the biopotential data in BLE (2.4 GHz) and a stable wireless communication link is established between a personal smartphone. Before displaying the real-time biopotential data, the EMG signal still contains considerable noise components which require further filtering (Figure 8b). Thus, a 12-tab IIR digital filter is implemented on the smartphone software for additional noise reduction (Figure 8d). Along with the right leg driving circuit and the dual analog–digital filter, most of the motion artifacts and interference noise is reduced down to a negligible level, making the proposed device suitable for an everyday use. Figure 9 displays the effect of noise reduction in frequency domain. 

**Figure 10:**
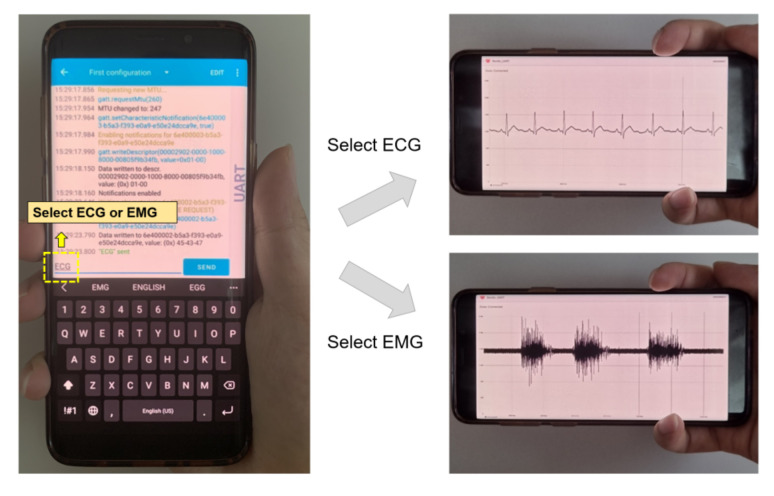
Mode selection and real-time signal monitoring from the host device.

Once the monitoring device is paired with BLE and the target physiological signal is selected, the device starts to capture the waveform. The information is displayed in a real-time plot on the personal Android device (Figure 10) and logged simultaneously as a file in the internal storage and synchronized to the network server. 

### 3.2. Accuracy of the Acquired Signal

**Figure 11:**
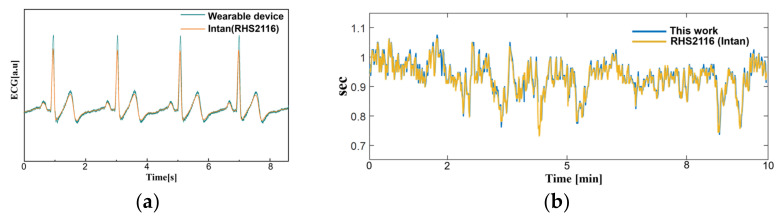
A comparison between this work and RHS2116 Stim/Recording System (Intan). (**a**) Time domain ECG acquired from this work and reference instrument at lead II orientation. (**b**) The R-R peak interval (heart rate) acquired for 10 min.

**Figure 12:**
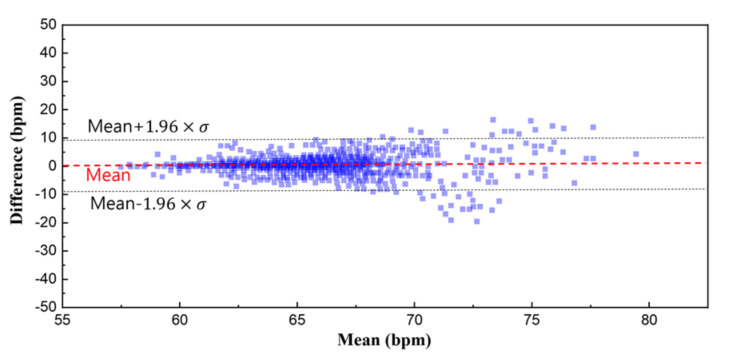
Bland–Altman plot of the heart rate. The overall signal coverage of this work shows an almost perfect overlay compared to the reference instrument within a difference of 0.2 ± 4.65 bpm.

In order to validate the signal quality of the proposed monitoring system, a reference physiological signal acquisition instrument (RHS2116 Stim/Record System, Intan) is used to compare the measurement. The reference instrument samples the biopotentials at 20 kSps with 16-bit resolution. Since the reference instrument is benchtop equipment, a short 10 min ECG recording session was analyzed for comparison. To remove interference between the two measurement instruments, electrodes from each instrument are placed side by side (approximately 10 mm away from each other), in alignment with each other. The transient physiological signals measured with both this work and the reference instrument are shown in Figure 11a. At a glance, the ECG measurement results show an almost perfect overlay of each other. The distribution of R-R peak interval for a 10 min recording session is shown in Figure 11b, which also show a close match. A Wilcoxon signed-rank test is performed to obtain a *p*-value of 0.133. Figure 12 shows Bland–Altman analysis of the signal coverage comparing the heart rate of both this work and the reference instrument. The signal quality results in a very close match with the RHS2116 recording system, showing a difference of less than 0.15 ± 4.65 bpm. 

**Figure 13:**
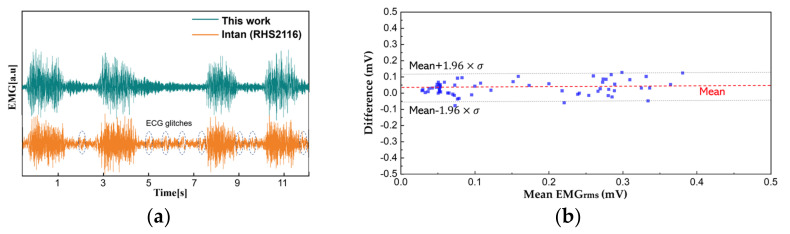
A comparison between this work and reference instrument. (**a**) Time domain EMG signals acquired from the right medial gastrocnemius muscle; (**b**) the Bland–Altman plot from EMG_RMS_.

The EMG comparison analysis is performed similarly. The electrodes are placed at the right medial gastrocnemius muscle with each electrode, for this work and the reference instrument, placed side by side (approximately 10 mm away from each other). The patient applies force to the muscle while standing. The transient EMG signals are measured as shown in Figure 13a. Since the analog signal amplification gain of the two-measurement method is different, we perform a back calculation to reconstruct the original signal. Discrepancy between the two results can be observed because the biopotentials are acquired from different locations within a muscle, but the overall EMG activity remains similar. A Bland–Altman analysis is performed from the root mean square values over an interval of 200 ms (Figure 13b). Considering the sEMG signals are obtained from different electrodes, the analysis shows close match with a difference of 0.031 ± 0.045 mV. 

### 3.3. Long-Term Monitoring and Data Analysis

**Figure 14:**
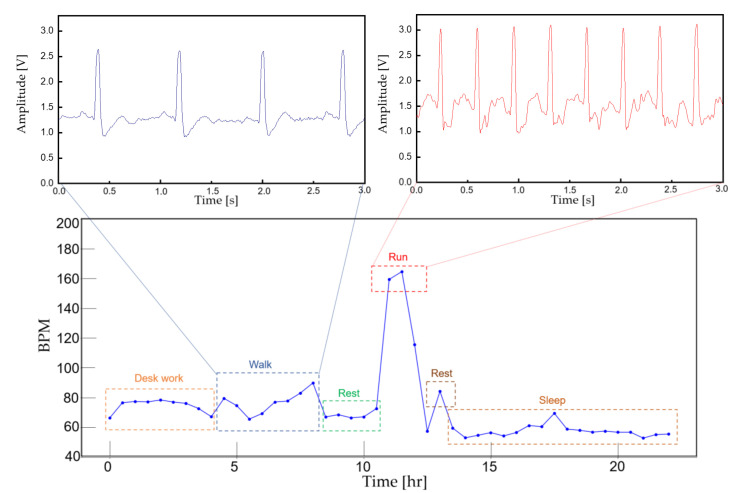
A continuous 30 min averaged BPM for 24 h. The actual ECG signal is displayed for walking and running activity.

**Figure 15:**
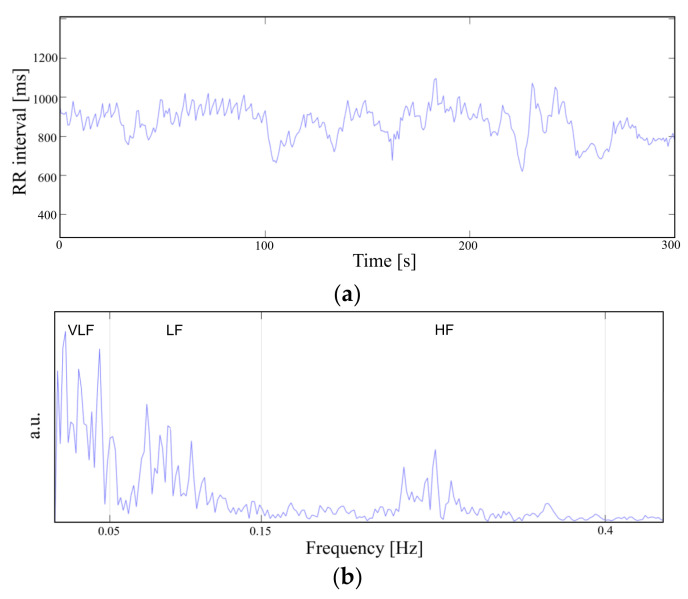
Heart rate variability in (**a**) time domain and (**b**) frequency domain.

**Figure 16:**
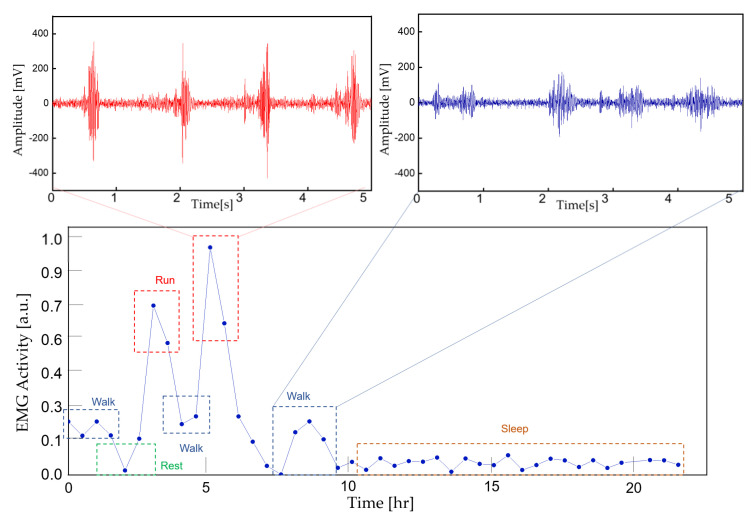
A continuous 30 min averaged EMG intensity for 24 h. The actual EMG signal is displayed for running and walking activity.

To demonstrate the feasibility of the device for daily use, the ECG and EMG waveforms were captured from a healthy male subject for a 24 h continuous monitoring session. The subject had a regular routine while wearing the device, including daily exercise, walk and sleep. Other than the patch electrode had to be attached to the skin for a prolonged period, the subject did not experience any noteworthy discomfort. The uploaded ECG data was further processed for an R-peak detection by comparing the gradient of the waveform and comparing it with a threshold value. A waveform smoothing process was performed to the calculated gradient values for noise rejection. Through the R-peak detection, a 30 min average BPM is plotted for the test session with sub-windows displaying the actual time-domain waveform during a running and walking session (Figure 14). A HRV is plotted during the rest session to demonstrate the fidelity of the monitoring device. The time interval between the R-peak is shown in Figure 15a with standard deviation of normal-to-normal interval (SDNN) and root mean square of successive differences (RMSSD) of 42.9 and 21.8, respectively. The frequency domain spectral analysis (Figure 15b) indicates a low frequency (LF), high frequency (HF) and LF/HR ratio of 77.7, 22.3, and 3.48, respectively. All HRV measurements indicate a normal and healthy condition. For the EMG session, the uploaded data were rectified and the envelop of the waveform was captured to calculate the EMG intensity. The normalized 30 min average of the intensity is plotted for the test session with sub-windows displaying the transient waveforms during a running and walking session (Figure 16). The feasibility test demonstrates that the physiological signal monitoring system is capable of capturing ECG and EMG signals during everyday activity without any noticeable artifact or interference. 

### 3.4. Device Lifetime

**Figure 17:**
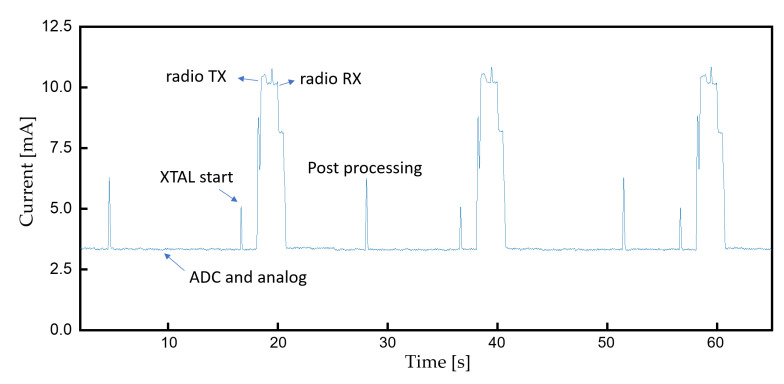
Current consumption during continuous monitoring.

The current consumption of the device at a 10 kSps sampling rate is measured as Figure 17 with an average of 4 mA. The device can continuously operate more than 58 h with a 235 mAh button cell battery. The cap of the resin assembly is easily removable for convenient battery replacement. The diameter and thickness of the assembly is 30 and 4.5 mm, respectively.

## 4. Conclusions

This study demonstrates a continuous ECG/EMG monitoring system for an everyday wearable application. The signal acquisition system accurately captures the biopotential and transmits the data to a smartphone for real-time monitoring and analysis. The application regarding signal monitoring is established on a personal smartphone to enhance the accessibility for elderly and handicapped patients. The connectivity between the cloud server also enables further data analysis and remote clinical support. The feasibility study results show that the device can effectively filter the interference signals and capture continuous ECG/EMG data during everyday activity. By preserving wireless connectivity to BLE-enabled devices, we anticipate our wearable physiological signal monitoring system as a healthcare tool for post-stroke and motor rehabilitation at-home environments where standard monitoring devices are not reachable.

## 5. Discussion and Future Work

In this work, we have demonstrated the capture of interference-free real-time physiological signals, presentation of a visual result to the patient, and delivery of the data to a dedicated server for further analysis. A comparison with a high precision instrument is presented, ensuring an accurate measurement. Although we have made notable improvements compared to the existing devices, there is further progress that needs to be worked on in order to deploy the system for practical use in the clinic with high impact.

- The current monitoring device has a ring hole and an adjustable band that the patient can tie onto their limb or neck. Although the rigid structure offers protection for the monitoring device, it can sometimes be uncomfortable to the patient. A flexible patch-type miniature monitoring device that be attached to the skin could be a solution.
- Feature extraction in the host device (smartphone) is necessary since it can sometimes be very difficult to spot a past event and the file size can be large in a prolonged recording session.
- Without a classification algorithm based on machine learning that can alert the patient or the physician, abnormal activity can easily go undetected unless a specialist monitors the data all the time. Hence, there is a need to integrate the current system with an algorithm that can effectively detect specific features, make accurate predictions, and alarm the patient or the physician [25,26].

## Figures and Tables

**Table 1:** Specification of the monitoring device circuit.

Symbol	Parameter	Value
Sampling Frequency	Sample per second	10^4^
A/D Resolution	Bit	8~12 (this work: 8)
V_DD_	V	1.8~3.3
Bandwidth (ECG)	Hz	0.34~41
Bandwidth (EMG)	Hz	40.17~727
Gain	V/V	1100
Communication Type	-	BLE
PCB Dimension	mm^3^	15 × 10 × 0.5
